# Adverse Effects of Systemic Immunosuppression in Keratolimbal Allograft

**DOI:** 10.1155/2012/576712

**Published:** 2012-02-28

**Authors:** M. Krakauer, J. D. Welder, H. K. Pandya, N. Nassiri, A. R. Djalilian

**Affiliations:** Department of Ophthalmology and Visual Sciences, Illinois Eye and Ear Infirmary, University of Illinois at Chicago, 1855 West Taylor Street, Chicago, IL 60612, USA

## Abstract

*Purpose*. Keratolimbal allograft (KLAL) is a treatment for limbal stem cell deficiency. One disadvantage is systemic immunosuppression to avoid rejection. Our purpose was to examine the adverse effects of systemic immunosuppression in KLAL. *Methods*. A retrospective case review of 16 patients with KLAL who received systemic immunosuppression consisting of a corticosteroid, an antimetabolite, and/or a calcineurin inhibitor was performed. Patients were monitored for signs, symptoms, or laboratory evidence of toxicity. *Results*. Eleven of 16 patients (68%) experienced an adverse effect. The average age of those with adverse effects was 43.5 years and without was 31.4 years. Ten of 11 patients (91%) had resolution during mean followup of 16.4 months. No serious adverse effects occurred. The most common included anemia, hyperglycemia, elevated creatinine, and elevated liver function tests. Prednisone and tacrolimus were responsible for the most adverse effects. Patients with comorbidities were more likely to experience an adverse effect (82% versus 20%, *P* = 0.036). *Conclusions*. KLAL requires prolonged systemic immunosuppression. Our data demonstrated that systemic immunosuppression did not result in serious adverse effects in our population and is relatively safe with monitoring for toxicity. In addition, we demonstrated that adverse effects are more likely in older patients with comorbidities.

## 1. Introduction

Limbal stem cell deficiency can develop following destruction of the limbal epithelial stem cells (e.g., chemical injury), or in conditions that impair the development or differentiation of the limbal stem cells (e.g., aniridia). The prevalence is unclear with only a fraction of patients with the above conditions going on to develop deficiency. In limbal stem cell deficiency, the cornea becomes covered by ingrowing conjunctival epithelium leading to recurrent epithelial breakdown, neovascularization, and scarring with subsequent loss of vision. Limbal stem cell transplantation is currently the main surgical treatment for visually disabling, total limbal stem cell deficiency. Keratolimbal allograft (KLAL) is the surgical procedure of transplanting cadaveric limbal grafts for patients with bilateral limbal stem cell deficiency, with a reported success rate of around 73% at 5 years [1].

The postoperative management of patients after KLAL is crucial to determining the long-term outcome of the procedure. Compared to conventional penetrating keratoplasty, limbal allografts are at higher risk for rejection mainly because the limbal area is vascular, and thus more accessible to the immune system, than the central cornea. Previous studies have demonstrated the importance of topical and oral immunosuppression in maintaining graft survival. In one series of aniridia patients treated with KLAL, the success rate was 90% in patients receiving systemic immunosuppression compared to 40% in patients treated with only topical immunosuppression [2]. 

The KLAL immunosuppression regimen is similar to that used in solid organ transplantation, where high rates of toxicity have been reported [3, 4]. The risks of immunosuppression must be balanced against the importance of the survival of the graft for functional vision in these patients. Holland and coworkers have recently demonstrated the relative safety of systemic immunosuppression in patients receiving KLAL and living-related conjunctival limbal allografts (lr-CLAL) [5]. Our purpose was to examine the adverse effects of systemic immunosuppression in patients with keratolimbal allograft.

## 2. Material and Methods

A retrospective case review was conducted on 16 patients (19 eyes) with documented limbal stem cell deficiency that underwent keratolimbal allograft at the University of Illinois at Chicago Medical Center (UIMC) between January 2006 and June 2009. Institutional Review Board approval was obtained. Written informed consent in accordance with the Declaration of Helsinki was obtained from all patients. The diagnosis of limbal stem cell deficiency was made based on clinical findings including loss of the limbal palisades, conjunctivalization of the corneal surface, superficial neovascularization, and nonhealing corneal epithelial defects. Prior to surgery, patients were evaluated by their primary care provider or an organ transplant specialist to determine their eligibility for systemic immunosuppression. All operations were performed by a single corneal surgeon (ARD) of the Cornea Service of UIMC. 

Postoperatively, all patients received prednisolone acetate 1.0% every 4–6 hours, gatifloxacin 0.3% (Zymar, Allergan, Irvine, CA) every 6 hours, and lubrication with nonpreserved artificial tears every 2 hours as needed. Once the corneal epithelial defect had healed, gatifloxacin 0.3% was discontinued and cyclosporine 0.05% (Restasis, Allergan, Irvine, CA) every 8 hours was started. Patients were examined for graft function and signs of rejection every 1-2 weeks for the first month and every month thereafter. Acute rejection episodes were managed with oral prednisone 1 mg/kg/day, prednisolone acetate 1.0% every 1-2 hours, and, in some cases, a 0.2 mL subconjunctival dexamethasone injection (40 mg/mL). 

Systemic immunosuppression was initiated immediately following surgery with regimens listed in Table 1. All patients were started on a prednisone taper starting at 1 mg/kg/day. In addition to prednisone, patients were started on tacrolimus 2–4 mg twice daily to yield whole blood 12-hour troughs of 5–10 ng/mL or cyclosporine (CsA) 3-4 mg/kg/day daily to a trough of 150–250 ng/mL; mycophenolate mofetil (MMF) 500–1000 mg twice daily or azathioprine 100–150 mg daily. Nine patients received tacrolimus plus MMF, and three patients received CsA plus MMF. One patient received tacrolimus plus sirolimus (Rapamune; Wyeth, Maidenhead, United Kingdom) 1-2 mg daily. Monotherapy with MMF, azathioprine, or CsA was used in 3 other patients. Treatment decisions were based on patient characteristics; for example, the oldest patient (81 years) was treated with prednisone and azathioprine alone, because it was felt that he did not require as potent immunosuppression as younger patients. The duration of treatment was determined based on ocular surface stability, defined as absence of inflammation, epithelial defects, and corneal neovascularization. Rejection was defined as presence of perilimbal injection, edema and infiltration of the graft leading to epithelial defects, and keratinization. 

In coordination with an internist, complete blood count, comprehensive metabolic profile, renal and liver profiles, fasting serum lipids, and fasting blood glucose were followed every 1-2 weeks for one month and then every month thereafter. A laboratory value was considered adverse if it occurred in two consecutive tests or if it prompted intervention. Leukocytosis while on prednisone was not considered an adverse effect, since it did not alter management or have a clear adverse consequence. Elevated intraocular pressure and cataract were not recorded as adverse effects and were attributed to the concurrent use of topical steroids. 

Prophylactic therapy was prescribed on a case-by-case basis. Four patients with a history of herpes simplex virus infection received valacyclovir (Valtrex) 500 mg twice daily for HSV prophylaxis. Three patients over 60 years received trimethoprim-sulfamethoxazole for *Pneumocystis carinii* prophylaxis. One patient that was known cytomegalovirus-negative received valganciclovir (Valcyte) 900 mg daily for CMV prophylaxis.

## 3. Results

Nineteen eyes of 16 patients aged 10–81 years (mean 42.6 years, SD = 20.0) were treated with KLAL and systemic immunosuppression and followed up for 4–39 months (mean, 16.4 months). The etiologies of limbal stem cell deficiency were aniridia (11 patients), chemical injury (2 patients), radiation therapy (1 patient), atopic keratoconjunctivitis (1 patient), and idiopathic (1 patient). Five patients had bilateral KLAL and 6 patients had repeat KLAL, 3 of which had their first procedure at an outside institution prior to this study. At last followup, 15 of 19 (79%) allografts were successful and 4 of 19 (21%) allografts had failed.

Ten of 16 patients (62%) had comorbidities. The most common comorbidities at the time of transplantation were hypertension and hyperlipidemia (4 patients each) followed by hyperglycemia/diabetes mellitus (3 patients), asthma/allergic rhinitis (2 patients), hypothyroidism (2 patients), fibromyalgia (1 patient), and migraine (1 patient). One patient had a history of elevated liver transaminases prior to enrollment (history of alcohol abuse), however, was cleared by their primary physician to be on immunosuppressive therapy.

The most common immunosuppression regimen was prednisone, MMF, and tacrolimus (9 patients). There was a wide variation in duration of therapy, which was based on the patient's response to therapy, graft viability, and whether the patient underwent a subsequent procedure. The general goal was to taper off prednisone within 3–6 months and to discontinue the other agents by 18–24 months. Four of 16 patients (25%) had repeat or bilateral KLAL which significantly extended the average duration of therapy. For example, those receiving a single procedure tapered to 2.5 mg of prednisone by an average of 6.7 months versus 15 months in those receiving multiple or repeat KLAL. Prednisone was tapered to 10 mg in 12 patients (75%) by 3 months. It was tapered to 2.5 mg in 8 patients (50%) by 6 months. Seven patients were completely tapered off all immunosuppressive medications without rejection.

Adverse effects are summarized in Table 2. Five patients (31.3%) experienced no adverse effects. Abnormal lab values were present in 7 patients (56%). Two diabetic patients had hyperglycemia that required additional treatment (18.8%). Two patients developed elevated creatinine (12.5%), one of which was a diabetic. Two other patients had elevated liver transaminases and hyperbilirubinemia (12.5%), one of which also had anemia and thrombocytopenia. One other patient developed anemia (6.3%) and one hypomagnesemia (6.3%). One case of anemia resolved with a multivitamin, and the other case resolved with vitamin B12. The abnormal liver function tests resolved after switching from azathioprine to MMF however, remained mildly elevated in the patient with possible alcohol abuse. One of the diabetic patients who developed hyperglycemia was treated with insulin and the other with additional oral hypoglycemic agents. In both cases, the glucose control returned to baseline upon prednisone taper. The elevated creatinine in one case returned to normal upon reducing the dosage of tacrolimus, however, remained mildly elevated in the diabetic patient until tacrolimus was completely tapered off. The case of hypomagnesemia resolved without intervention. Overall, the only patient who had persistent laboratory abnormalities was a patient with possible alcohol abuse who was lost to followup.

Clinical adverse effects were seen in 4 patients (25%): One had hypertension (6.3%), 1 had headaches and tremors (6.3%), 1 had acne (6.3%), 1 had HSV keratitis (6.3%), and 1 had *Candida albicans* corneal ulcer (6.3%). The case of headaches and tremors resolved with decreasing the dose of tacrolimus. HSV keratitis was treated successfully with oral acyclovir. The acne persisted after prednisone taper and finally resolved upon discontinuing tacrolimus. The ulcer resolved with therapeutic keratoplasty. 

The average age of the 11 patients with adverse effects was 43.5 years (range 10.8–81.6, SD = 20.8) and for the 5 without adverse effects was 31.4 years (range 19.3–40.7, SD = 20.5). Nine of 11 (82%) patients experiencing adverse effects had comorbidities, whereas only 1 of 5 (20%) patients without adverse effects had comorbidity. Comorbidities included hypertension, diabetes, liver disease, hyperlipidemia, kidney disease, and other chronic medical illnesses.

## 4. Discussion

KLAL is the main therapeutic option for bilateral, total limbal stem cell deficiency. Due to the vascularity of the limbal area, topical, and systemic multidrug immunosuppression is necessary to allow graft survival. Based on the experience of solid organ transplantation, multidrug regimens are preferred since they achieve a higher level of immunosuppression with lower doses, and thus lower toxicity, than individual medications used as monotherapy.

We present here a series of 16 patients who underwent KLAL and systemic immunosuppression. Previously, the adverse effects of systemic immunosuppression with KLAL have not been reported in such detail. Based on prior studies by Holland and coworkers and our experience, our preferred regimen is prednisone, mycophenolate mofetil, and tacrolimus; nine of the patients in this series were on this regimen. There was a wide variation in duration on each medication, which was based on the patient's response to therapy, that is, signs of rejection or favorable response, as well as whether the patient underwent a subsequent procedure, which required longer duration. The general goal was to taper off prednisone within 3–6 months and to discontinue the other agents by 12–18 months. In order to achieve a more rapid taper of prednisone, we have recently begun to use more potent topical steroids such as difluprednate (Durezol, Alcon, Irvine, CA). Our current goal is to discontinue the oral steroids by 3 months. Antimicrobial prophylaxis is used on a case-by-case basis.

Eleven (69%) patients experienced an adverse effect to systemic immunosuppression, and 10 of those patients (91%) experienced resolution of the adverse effect during followup. There were no life-threatening complications. The most serious adverse effect was one case of *Candida albicans* corneal ulcer. The corneal ulcer was attributed to the patient's preexisting ocular surface disease, while the particular infectious agent was most likely due to topical steroids. 

Patients experiencing an adverse event were on average older than the group without adverse events (43.5 versus 31.4 years, *T*-test *P* < 0.05). In addition, those with significant comorbidities, including diabetes, hypertension, kidney disease, liver disease, depressed immune systems, and hyperlipidemia, were more likely to experience an adverse event (82% versus 20%, *P* = 0.036). The most adverse events were seen in patients with diabetes. For example, one diabetic had problems with blood sugar, kidney function, and liver enzymes. In addition, one patient with alcohol abuse had elevated liver enzymes, blood sugar, and macrocytic anemia, making him a poor candidate for systemic immunosuppression. We conclude from this data that KLAL with systemic immunosuppression is relatively contraindicated in older patients and those with significant comorbidities. However, in young and healthy patients, it is rare to experience serious adverse effects from systemic immunosuppression in KLAL, making them great candidates for the procedure. 

Holland and coworkers (2007) have already demonstrated in an age-matched study that ocular surface transplant recipients experience significantly fewer adverse effects to immunosuppression when compared to renal transplant recipients [6]. Their group was the first to suggest that the patient population receiving ocular surface transplants such as KLAL may be much more tolerant of systemic immunosuppression, likely owing to the relative abundance of comorbidities in other solid organ transplant recipients including hypertension, diabetes, kidney disease, and liver disease. A recent study by Liang et al. demonstrated that only 2 of 10 (20%) patients on combined mycophenolate mofetil and tacrolimus experienced adverse effects, which included hypertension, hyperbilirubinemia, and GI symptoms [7].

Similar data has been demonstrated in the use of immunosuppression for other ocular diseases. In one study of over 2000 patients receiving immunosuppression for ocular inflammation found no increased mortality from cancer in patients receiving antimetabolites (methotrexate, azathioprine, and mycophenolate mofetil) or T-cell inhibitors (CsA) for eye disease [8]. A study of 373 patients taking CsA for ocular inflammation reported only 13% stopping treatment for an adverse effect, which were most commonly renal toxicity or hypertension. They also demonstrated a severalfold increase in discontinuation in patients greater than 55 years of age compared to younger patients [9]. In a similar study, of 236 patients treated with MMF, only 12% withdrew due to adverse effects, with most of these resolving after discontinuation [10].

In corneal transplant surgery, a study of 43 corneal transplant patients receiving tacrolimus found adverse effects in 24 of 43 (56%) including 4 with lab abnormalities and 17 with symptoms such as headache, malaise, GI symptoms, paresthesia, or insomnia. One patient developed pancreatitis [11]. Another study showed that 6 of 29 (21%) receiving MMF and 11 of 27 (41%) receiving CsA experienced an adverse effect at doses similar to our regimen, most commonly hepatotoxicity (3 and 2, resp.) and hypertension (3 and 0, resp.). While on CsA, one patient developed Hodgkin's lymphoma and one patient had a recurrence of acoustic neuroma [12]. Another corneal transplant study showed that 22 of 49 (44.9%) receiving CsA experienced an adverse effect, including 10 with lab abnormalities, 4 with hypertension, and 6 with symptoms such as nausea/vomiting, backache, or tremors [13]. Looking specifically at limbal-keratoplasty utilizing CsA and/or MMF, one study of 48 patients showed elevated creatinine in 9, liver enzymes in 6, and blood pressure in 5 patients [14]. Another study showed that 1 of 3 patients receiving CsA had elevated creatinine [15]. In a study mentioned previously, MMF, tacrolimus, and prednisone were used in 23 cases of KLAL and lr-CLAL and resulted in 0 serious adverse events, 4 temporary creatinine elevations, and 2 transient transaminase elevations [5]. In contrast, Ekberg et al. (2007) showed significant side effects in 43–53% of renal transplant patients receiving cyclosporine and tacrolimus, including 1–2.7% with new-onset diabetes, 25% with opportunistic infections, 9 patients developed cancer, and 4 deaths were considered to be caused by an adverse effect of systemic immunosuppression [3]. 

Our study demonstrates that with close monitoring for signs, symptoms, and laboratory abnormalities caused by drug toxicity, systemic immunosuppression in KLAL is generally safe. Patient selection and expectation is critical; it is expected that patients with significant comorbidities such as diabetes and older age will be more likely to experience adverse effects, and, therefore, caution must be exercised in such patients.

## Figures and Tables

**Table 1:** Oral immunosuppression therapy regimen.

Class	Starting dose	Medication	Number of patients	Avg. duration (months)
Corticosteroid	1 mg/kg/day	Prednisone	16	12.8 (Median:10, Range: 3–25)
Calcineurin inhibitor	3-4 mg/kg/day	Cyclosporine	4	13.7 (Range: 4–19)
	2–4 mg BID	Tacrolimus	10	17.8 (Range: 5–25)
Antimetabolite	100–150 mg/day	Azathioprine	2	5.7 (Range: 1–16)
	500–1000 mg BID	Mycophenolate mofetil	12	16.9 (Range: 5–39)
IL-2 inhibitor	2 mg/day	Sirolimus	1	22

**Table 2:** Adverse effects of systemic immunosuppression.

Frequency (Number of patients)	Adverse effect (level)	Adverse effect status
2	Elevated transaminases and hyperbilirubinemia (8.6, 3.8)	Resolved by switching azathioprine to mycophenolate; not completely resolved possibly from alcohol abuse
2	Elevated creatinine (1.5, 1.6)	Resolved after reducing/discontinuing tacrolimus
2	Hyperglycemia	Treated with insulin; resolved by increasing oral hypoglycemic medication
2	Anemia (Hgb 11.9, 10.9)	Resolved with vitamin B12; resolved with multivitamin
1	Hypomagnesemia (1.6)	Self-resolved
1	Worsening hypertension	Resolved with increased medication
1	Thrombocytopenia (89)	Not yet resolved, possibly from alcohol abuse
1	Acne	Resolved after tapering off prednisone and tacrolimus
1	Headache, tremors	Resolved by decreasing tacrolimus
1	Candida corneal ulcer	Resolved
1	HSV keratitis	Resolved with oral acyclovir
1	(1.6)	Self-resolved
